# Mobile Computing with a Smart Cricket Ball: Discovery of Novel Performance Parameters and Their Practical Application to Performance Analysis, Advanced Profiling, Talent Identification and Training Interventions of Spin Bowlers

**DOI:** 10.3390/s21206942

**Published:** 2021-10-19

**Authors:** Franz Konstantin Fuss, Batdelger Doljin, René E. D. Ferdinands

**Affiliations:** 1 Chair of Biomechanics, Faculty of Engineering Science, University of Bayreuth, D-95440 Bayreuth, Germany; 2 Smart Products Engineering Program, Swinburne University, Melbourne, VIC 3000, Australia; bdoljm@swin.edu.au; 3 Discipline of Exercise and Sports Science, School of Health Sciences, Faculty of Medicine & Health, University of Sydney, Sydney, NSW 2141, Australia; edouard.ferdinands@sydney.edu.au

**Keywords:** smart cricket ball, performance analysis, mobile computing, skill performance, precession, gyroscope, spin rate, torque, wrist-spin, finger-spin, efficiency

## Abstract

Introduction: Profiling of cricket bowlers is performed with motion analyses systems that require the placement of markers on the bowler’s body and on the ball. Conventional smart balls such as cricket and baseballs provide only one speed and one spin rate datum at the release point, which is insufficient for biomechanical profiling. Method: In this study, we used an advanced smart cricket ball that measures the angular velocity at 815 Hz and calculates four further physical performance parameters (resultant torque, spin torque, power and angular acceleration) and five new skill parameters (precession, normalised precession, precession torque, efficiency and ratio of angular acceleration to spin rate), which we used for profiling and talent identification of spin bowlers. Results: The results showed that the spin rate is a function of physical (torque) and skill proficiency, namely how efficiently the torque is converted to angular velocity rather than being wasted for precession. The kind of delivery also influences the efficiency, as finger-spin deliveries were less efficient than wrist-spin ones by 6.8% on average; and topspin deliveries were generally more efficient than backspin ones by 15% on average. We tested three bowlers in terms of physical and skill performance during a 10-over spell, revealing that some parameters can improve or decline. When profiling a topspinner, we detected from the performance parameters a lower skill performance than expected, because there was an initial arm motion for backspin delivery before releasing the ball with a topspin. After training intervention, the skill parameters improved significantly (the efficiency increased from 39% to 59%). Conclusions: The advanced smart cricket ball is a classic example of mobile computing for sport performance analysis that can conducted indoors as well as outdoors, generating instant data from 10 performance parameters that provide critical feedback to the coach and bowler.

## 1. Introduction

Spin bowlers impart a torque to the cricket ball with their fingers to spin the cricket ball with a high rate of rotation (i.e., spin rate). In the field of play, it has been visually observed that a spinning ball can produce two effects in favourable conditions: swerve during flight, and lateral and vertical deviation off the pitch after the bounce [1,2]. These characteristics of a spinning ball can deceive the discriminative perception of batsmen so that they make inappropriate shot selections that can lead to their dismissal. If an erroneous perception in the deviation of the ball’s direction off the pitch occurs when it is less than 200 ms away from the batsmen, then the batsmen will not have sufficient time to execute a movement compensation [3]. In addition, any deviation in the ball’s flight prior to landing, a phenomenon called swerve resulting from the Magnus force [4,5] will further act to confound the perception of the batsman. Hence, skill acquisition researchers have used visual occlusion techniques to study the batsman’s ability to discriminate between spin bowling deliveries [6,7,8]. Since elite batsmen are better at discriminating between the types of spin deliveries by using advance cue utilisation [8], spin bowlers must carefully execute their variation deliveries, using them sparingly and skilfully disguising the mechanical differences between them.

The traditional assessment of spin bowling performance in a scientific set-up relies on motion analysis, which is usually performed in an indoor biomechanics laboratory. Typically, for technical analysis, a spin bowler will be asked to deliver a series of balls with a maximal spin rate on an artificial pitch while being fitted with numerous small retroreflective markers (usually more than 50) on the key bony landmarks, enabling a full body representation of motion using joint coordinate systems [9,10]. In addition, the ball will be fitted with a minimum of three markers to define a local coordinate system for the ball, so that both linear and spin angular velocity can be measured. For an analysis of spin bowling performance outcomes that are independent of technique, only the cricket ball needs to be tracked. By calculating the three-dimensional spin angular velocity vector, researchers have been able to calculate the rate of spin and estimate the direction of the Magnus force [4,5]. The spin rate is often considered as a general measure of a spin bowler’s skill ability, but a more complete evaluation of proficiency must consider a bowler’s capacity to change the orientation of the spin angular velocity vector. A skilful spin bowler can vary the orientation of the spin angular velocity vector to change the direction of Magnus force applied to the ball. By such means, the cricket ball can swerve in flight, both laterally and vertically. Moreover, if swerve is followed by a lateral deviation off the pitch on a “good length” that was not deciphered by the batsman, then it is unlikely that movement compensation of the bat will occur in time to successfully strike the ball, a scenario that will increase the chance of a dismissal.

Smart sports equipment came into fashion in the 2000s [11,12], and, with improvement of electronics and battery technology (e.g., miniaturisation), paved the way for smart wearable devices. It is nowadays an inherent part of the digital revolution in sports. The first smart cricket ball ever was developed by Fuss et al. [13,14] in late 2011, at the time when the first high-speed rate gyro was introduced (ADXRS649, Analog Devices, Wilmington, MA, USA) with a measurement range of ±20,000°/s (350 rad/s, 55.56 rps), extendable to ±50,000°/s. This ball was used subsequently for research, to understand the dynamics of bowling [13,14,15,16]. The ball was finally improved in 2014 [17] and upgraded to wireless data transfer, inductive charging, and control via a smartphone. The developers, then at RMIT University, joined forces with the University of Sydney for an extended investigation of spin bowling [18,19,20,21,22].

The first commercial smart cricket ball was introduced by Kookaburra in 2019/2020, measuring the speed and spin rate at the release point and before the bounce, and the speed after the bounce [23]. Owing to the lack of high-speed gyros, the spin rate is estimated from the frequency of the magnetometer signal during the flight of the ball [24]. This commercially available ball was developed “*to offer a fun, engaging and educational platform that lets players, coaches and fans engage with the sport on a whole new level*” [25], while [26] claims that this “*Smart Cricket Ball measures your bowling performance*”.

A fast bowler’s performance is not only determined by the speed of the ball, but also the ability to cause a deviation in ball flight, whether it be through conventional swing, reverse swing, or contrast swing [27,28].

In spin bowling, the analogue parameters are the spin rate [2] and the swerve during flight [4,5]. However, in spin bowling, there are also a large number of different delivery types that occur within each of the traditional classes of wrist-spin and finger-spin bowling, some of which are significantly more difficult to bowl than others. Therefore, the objective of this research is to show how the smart cricket ball can quantify both skill and physical performance in spin bowling.

The aim of this paper is to explain mathematically the different performance parameters obtained from our high-quality smart cricket ball, as well as to exemplify the application of these performance parameters by means of four different studies.

## 2. Materials and Methods

### 2.1. Development and Specifications of the Smart Cricket Ball

**Figure 1:**
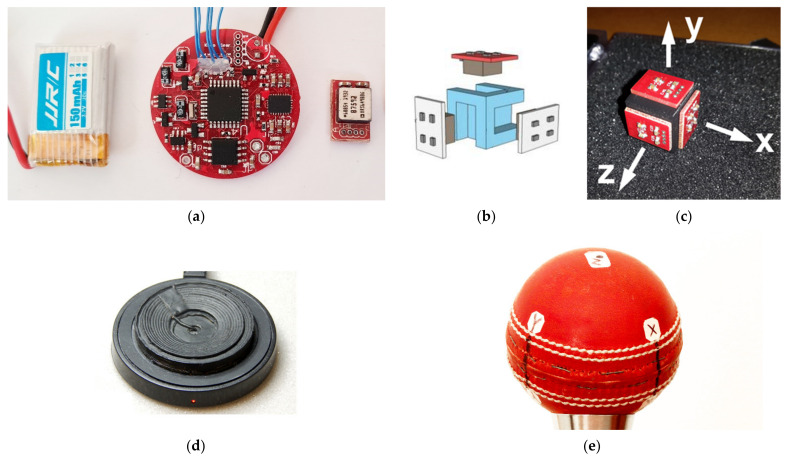
The smart cricket ball and its components; (**a**) from left to right: battery, circular PCB, sensor on breakout board; (**b**) sensor assembly; (**c**) assembled sensors on carrier; (**d**) inductive charging dock; (**e**) smart cricket ball.

For this study, we used the smart cricket ball (Figure 1) developed by Doljin and Fuss [17] together with the analysis software written in Python 2.7. The ball has the same properties (mass 160 g, leather surface) of a standard cricket ball. The cover was constructed from the leather skin of a commercially available cricket ball and a CNC-machined Nylon N6 shell, that was internally padded with closed-cell foam material to absorb the shock and vibrations during the impact.

We used three single-axis high-speed gyroscope sensors (ADXRS649, Analog Devices, Wilmington, MA, USA) to measure the spin rate of the cricket ball throughout the bowling action and the flight of the ball up to a maximum angular velocity of ±20,000°/s (350 rad/s, 55.56 rps). The main advantage of the ADXRS649 sensor is a quad-differential MEMS layout [29] that cancels out the effects of vibrations and linear acceleration. The sensors, mounted on breakout boards, were aligned orthogonally on a 3D-printed carrier.

A 150 mAh lithium battery provides continuous power to the electronics circuit that measures and records the spin rate of the ball. The battery can supply sufficient power for recording 80 continuous deliveries with a single charge. The power circuit utilises Qi charging technology to transfer the power wirelessly and inductively to the smart ball. The coil for inductive charging is sandwiched between the leather cover and the plastic shell.

In addition to the sensors and the power supply, the electronics consists of a 64 MB flash memory, an 8-bit RISC single-chip microcontroller, a capacitive touch switch, and a Bluetooth transceiver installed on the motherboard which stores the data sampled at 815 Hz and wirelessly transfers it to remote devices at 115,200 b/s (such as smartphone, tablet, and laptop, etc.). The motherboard (circular PCB, Ø 31 mm, Figure 1a) and small breakout sensor PCBs (13 × 9 mm (Figure 1a,c) were designed using Autodesk Eagle CAD software.

After assembly of the electronics items, the entire electronics unit was potted with epoxy resin and anchored to the closed-cell foam layer with strips of double-sided adhesive tape. The outer side of the foam layer was equally taped to the inner side of the nylon shell to prevent any movement between the components. The potted electronics unit was aligned to the ball’s coordinate system during the assembly of the ball.

To align the sensors’ coordinate system SCS to the ball’s coordinate system BCS, we CNC-machined an aluminium housing for the ball, made of two half cubes (part A and B) with hemispheric cavities of 40 mm radius. The smart ball was placed into hemisphere A which allowed for aligning the plane of the seam precisely to the plane where the two parts were joined together. The alignment process was achieved by small screws holding the seam in place, at the BCS axes of: +x, –x, +y, –y. The CNC-machining process ensured the orthogonality of these axes. Furthermore, the +z axis was represented by a 5th screw, drilled slightly into the north pole of the cricket ball. The alignment of the ball to its housing was similar to the process of attaching a halo frame to a skull for the purpose of stereotactic surgery. When joining part B to part A with screws, drill shafts were inserted at the junctions into pre-machined bore holes, aligned to x- and y-axes of the BCS, and at part B aligned to the z-axis. These shafts that fitted into a drilling machine allowed the precise rotation of the smart ball about the three axes of the BCS. By means of the small screws, the coordinate system was clearly indicated on the leather surface of the ball. As the potted electronics unit (and thus the SCS) was aligned manually, i.e., only approximately, to the BCS during the assembly of the ball, the angular velocity vectors (of the SCS) recorded by the smart ball, when rotating the ball about the three axes of the BCS, were not precisely aligned to the three BCS axes. This means that if the housing including the ball were rotated about +BCSx, then the components *ω_y_* and *ω_z_* of the angular velocity *ω* were unequal zero.

We aligned the SCS to the BCS by (1) rotating the SCS about +BCSy so that SCS-X lies in the plane of the seam and SCS-Xz becomes 0; (2) then rotating the SCS about +BCSz so that SCS-X = BCSx; and (3) finally, rotating the SCS about +BCSx so that SCS-Y lies in the plane of the seam and SCS-Yz becomes 0. We implemented the angles of the three rotation steps into the software routine and verified the correct alignment by rotating the ball again about the three axes of the BCS. This exercise was repeated every two months to ensure that the coordinate system’s alignment did not change.

Subsequently, we tested the orthogonality of the aligned SCS by calculating the angles between the three angular velocity vectors resulting from the preceding verification process. The orthogonality is affected by the mounting of the gyroscopes on the 3D-printed carrier. If the angle *ψ* between two gyroscopes is unequal 90°, and the angular velocity component of gyro X, namely *ω_x_* is defined as the reference, then *ω_y_* measured by gyro Y is underestimated by the cosine of |90°–*ψ*|. An underestimation or error of 1% or ¼% that results from arccos(1–0.01) or arccos(1–0.0025) corresponds to a right angle deviation by 8.11° or 4.05°, respectively. Errors of the angular velocity of 1% do not make a difference in performance and are therefore acceptable. The same is true for further performance parameters calculated from the angular velocity such as the torque (same error as the angular velocity) or the power (twice the error of the angular velocity). The angles of the SCS in the xy–, xz–, and yz–planes of the BCS were 87.87°, 91.69°, 90.03°, respectively, which correspond to errors of 2.13°, 1.69°, 0.03°, respectively.

The angular velocity measured by each analogue gyro was recorded by the microcontroller with a 10-bit resolution. The null bias (1.65 V at the ADC input) at zero angular velocity was not necessarily equal to 1023/2 = 511.5 ASCII and was determined statically at 0 rad/s. The average static ASCII data of each gyro were used as the baseline and subtracted from the ASCII data. The resulting ASCII data differential was divided by the sensor calibration factor to obtain the angular velocity in revolutions per second (rps). A factor of 7.2 corresponding to 1 rps was found from calibration [14].

### 2.2. Performance Parameters

A total of 10 different performance parameters—physical and skill performance (Table 1) were analysed and are explained subsequently. In particular, the skill parameters are newly discovered and have not been used before nor applied as performance parameters.

(a) Angular velocity *ω_R_* of the ball: the spin rate results directly from the *x*,*y*,*z* angular velocities (*ω_x_*, *ω_y_*, *ω_z_*) measured by the three gyros incorporated into the ball,

(1) $$\omega_{R}=\sqrt{\omega_{x}{}^{2}+\omega_{y}{}^{2}+\omega_{z}{}^{2}}$$

The spin rate is expressed in rps in this paper (revolutions per second; 1 rps = 2π rad/s; SI unit: rad/s). Subsequently, *ω_R_* will be denoted simply by *ω*, as in the course of deriving further parameters, new subscripts of *ω* will be used, e.g., 0 for the initial condition, *t* for time, min for minimum, etc.

(b) Angular acceleration *α* (SI unit: rad/s^2^), the time derivative of the angular velocity,

(2) $$\alpha_{xyz}=\frac{d\omega_{xyz}}{dt}$$

and

(3) $$\alpha_{R}=\sqrt{\alpha_{x}{}^{2}+\alpha_{y}{}^{2}+\alpha_{z}{}^{2}}$$

is produced mainly by muscles crossing the finger, wrist and forearm joints during the delivery of the ball before release.

(c) The resultant torque *T_R_* (SI unit: Nm) is calculated from Euler’s equations of motions, which are functions of angular velocity and acceleration.

(4) $$T_{xyz}=I_{xyz}\alpha_{xyz}+\left(I_{zxy}-I_{yzx}\right)\omega_{yzx}\omega_{zxy}$$

where *I* denotes the moment of inertia of the ball, and *I_x_* ≈ *I_y_* ≈ *I_z_*.

*T_R_* results from

(5) $$T_{R}=\sqrt{T_{x}{}^{2}+T_{y}{}^{2}+T_{z}{}^{2}}$$

**Figure 2:**
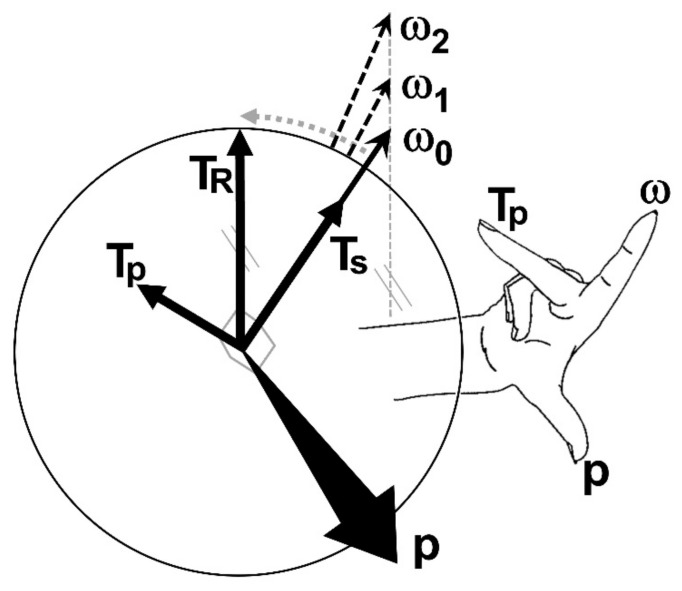
Basic principle of precession; **ω** = angular velocity vector (the subscripts refer to different times *t*, i.e., 0 at *t* = 0, etc.), **T_R_** = resultant torque vector, **T_s_** = spin torque component (same direction as **ω**), **T_p_** = precession torque component (perpendicular to **T_s_** and **ω**), **p** = precession vector (perpendicular to the **T_R_**-**ω** plane); the vectors **p**, **ω**, and **T_p_** are perpendicular to each other and follow the right-hand rule (**P-S-T rule**; S denotes the spin rate **ω**); the curved and dashed grey arrow indicates the movement of the **ω**-vector into the **T_R_**-vector, where the **ω**-vector’s magnitude increases continuously as it is accelerated by **T_s_**; note that the vertical dashed grey line connecting the arrow tips of the **ω**-vectors is parallel to the **T_R_**-vector, to satisfy Equation (22).

**Figure 3:**
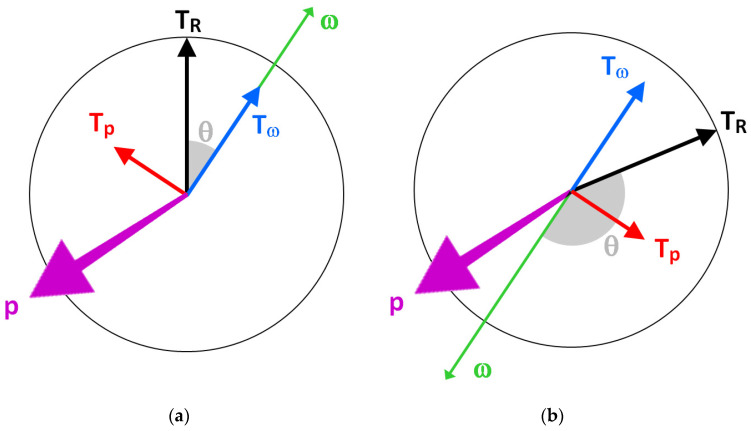
Principle of the two torque components: the torque vectors **T** and the angular velocity vector **ω** are located in the plane of the circle; **T_R_** is the resultant of the spin torque **T_s_** and the precession torque **T_p_**; **T_s_** is parallel to **ω**; **T_p_** is perpendicular to **ω**; θ is the angle between **T_R_** and **ω**; the precession vector **p** is perpendicular to the plane of the circle (pointing out of this plane); the sequence of the vectors **p**, **ω**, and **T_p_** follows the right-hand rule (comparable to the x,y,z axes of a coordinate system); (**a**): θ < 90° so that **T_s_** increases the magnitude of **ω**; (**b**): θ > 90° (fast bowling) so that **T_s_** reduces the magnitude of **ω** until θ = 90°.

(d) The resultant torque vector **T_R_** has two components (Figure 2 and Figure 3), the spin torque **T_s_** and the precession torque **T_p_**. **T_s_** and **T_p_** are perpendicular to each other; **T_s_** is parallel to the **ω**-vector; **T_p_** is perpendicular to the **ω**-vector. All four vectors are located in an instantaneous plane defined by **T_R_** and **ω**.

(6) $$T_{R}=\sqrt{T_{s}{}^{2}+T_{p}{}^{2}}$$

(7) $$T_{s}=T_{R}\cos\theta$$

(8) $$T_{p}=T_{R}\sin\theta$$

where *θ* is the angle between **T_R_** and **ω**

(9) $$\theta =\cos^{-1}\frac{\left|\omega \cdot T_{R}\right|}{\left|\omega \right|\left|T_{R}\right|}$$

**Figure 4:**
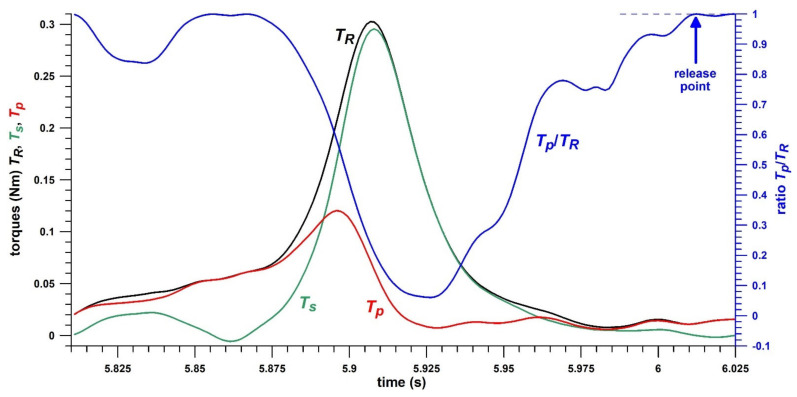
Torques against time; *T_R_* = resultant torque, *T_s_* = spin torque, *T_p_* = precession torque; the release point is determined from *T_p_*/*T_R_* reaching unity.

*T_p_* equals zero if *θ* = 0. It is evident that only *T_s_* produces the change of spin rate or angular velocity (thereby causing angular acceleration), whereas *T_p_* causes the precession of the spin axis vector **ω**. The precession torque component **T_p_** of the resultant torque **T_R_** is therefore a lost torque that could otherwise contribute to physical performance in terms of spin rate and angular kinetic energy. However, **T_p_** is required to move the spin axis vector **ω** into the torque vector **T_R_** [15], as **ω** always follows the (moving) **T_R_**. This becomes strikingly apparent in fast bowling, where the motion of the arm imparts topspin on the ball, whereas the fingers impart a backspin during the release of the ball. Therefore, the spin axis vector has to change its direction from pointing to the left (topspin) to pointing to the right (backspin) and move from one hemisphere of the ball to the other one (Figure 3), which is enabled by the precession torque *T_p_*. The torques against the time are shown in Figure 4.

(e) The precession *p* is calculated from:

(10) $$p=\frac{T_{R}\sin\theta }{\omega I}=\frac{T_{p}}{\omega I}$$

The unit of the precession *p* is rad/s when using SI units in Equation (10). Alternatively, *p* can be calculated directly from the angle *φ* between two consecutive spin axes times the data sampling frequency *f*:

(11) $$d\theta =\cos^{-1}\frac{\left|\omega_{t}\cdot \omega_{t+dt}\right|}{\left|\omega_{t}\right|\left|\omega_{t+dt}\right|}$$

and

(12) $$p=\frac{d\theta }{dt}=d\theta^{}f$$

(f) The normalised precession *p_n_* (SI unit: rad; unit used in this publication: degrees), normalised to *T_R_* and *ω*, results from solving Equation (10) for *θ* [18]:

(13) $$p_{n}=\theta =\sin^{-1}\frac{p\omega I}{T_{R}}$$

**Figure 5:**
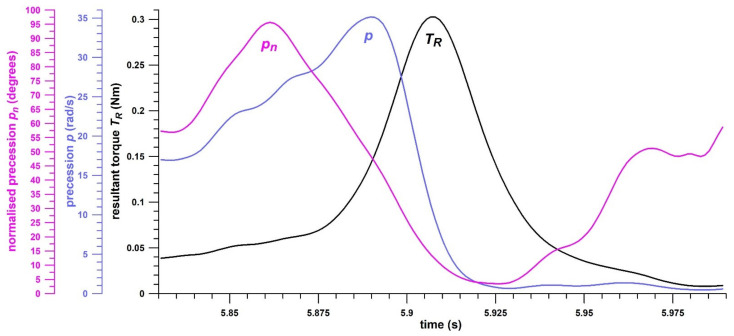
Normalised precession *p_n_* (= *θ*, the angle between the spin rate vector **ω** and the torque vector **T_R_**), precession *p*, and torque *T_R_*, against time, exemplifying the sequence of peak data; note that *p_n_* (= *θ*) drops to 3.5° after the *T_R_* peak.

The *p_n_*, expressed as the angle *θ*, is the angle between the spin vector **ω** and the torque vector **T_R_** at the instant when the **ω**-vector commences its major move into the **T_R_**-vector (Figure 2, Figure 3 and Figure 5). This is usually identified from the *p_n_*-peak before the *p*-peak. As *p_n_* values can be larger than 90 degrees, it is advisable to calculate *p_n_* directly from the angle between **T_R_** and **ω**-vectors from Equation (9). From Equation (10) it is evident, that the precession *p* is directly proportional to *T_R_* and the reciprocal of *ω*. Dividing *p* by *T_R_* and multiplying it by *ω* therefore normalises *p* and yields *p_n_*, as seen in Equation (13).

(g) The relation of *θ* (= *p_n_*), *ω*, *T_s_*, *T_p_*, and *p* is explained as follows:

*T_s_* generates the angular acceleration *α* of the spinning object, which in turn changes its angular velocity *ω*.

(14) $$-\alpha =-\frac{d\omega }{dt}=\frac{T_{s}}{I}=\frac{T_{R}\cos\theta }{I}$$

After rearranging terms 2 and 4 of Equation (14)

(15) $$\frac{I}{dt^{}\;T_{R}}=-\frac{\cos\theta }{d\omega }$$

*T_p_* generates the precession *p* of the spinning object, which tilts the **ω**-vector.

The precession is defined by Equations (10) and (12) which, after rearranging, yield:

(16) $$\frac{I}{dt^{}\;T_{R}}=\frac{\sin\;\theta }{d\theta^{}\;\omega }$$

Equating Equations (15) and (16)

(17) $$-\frac{\cos\theta }{d\omega }=\frac{\sin\theta }{d\theta^{}\;\omega }$$

and rearranging yields:

(18) $$\cot\theta^{}d\theta =-\frac{1}{\omega }d\omega$$

Note that the ordinary differential Equation (18) is independent of *T_R_*, d*t* and *I*.

Integrating both sides of Equation (18) yields:

(19) $$\log\left(\sin\theta \right)-\log\left(\sin\theta_{0}\right)=-\log\left(\omega \right)+\log\left(\omega_{0}\right)$$

where the integration constants correspond to the initial conditions; log denotes the natural logarithm; and sin(*θ*) and *ω* must be positive. Rearranging Equation (19) further results in:

(20) $$\log\left(\sin\theta \right)=-\log\left(\omega \right)+\log(C)$$

a linear equation with unity gradient and intercept log(*C*). As log(*ω*_0_) + log(sin *θ*_0_) = log(*ω*_0_ · sin *θ*_0_), *C* = *ω*_0_ · sin(*θ*_0_), the product of the initial conditions.

If both components of *C* are unity, i.e., *ω*_0_ = 1 rad/s and *θ*_0_ = π/2 rad = 90°, then *C* = 1 and log*C* = 0 so that Equation (20) collapses to sin(*θ*) = 1/*ω*, a power law relationship with exponent of –1 (reciprocal function).

Anti-logarithmising Equation (19) yields:

(21) $$\frac{\sin\theta }{\sin\theta_{0}}=\frac{\omega_{0}}{\omega }$$

Therefore,

(22) $$\omega \sin\theta =\omega_{0}\sin\theta_{0}=C$$

Equation (22) shows that the product *ω* · sin(*θ*), is a constant, independent of *T_R_*, d*t*, *I*. If *θ* → 0, then sin(*θ*) → 0 and *ω* → ∞. In other words, **ω** can never move into, and reach, **T_R_** entirely [16]. The product *ω* · sin(*θ*) corresponds to the distance between **T_R_** and a line parallel to **ω** where the endpoints of consecutive **ω**-vectors are located (Figure 2).

Solving Equation (22) for *θ*

(23) $$\theta_{1,2}=\sin^{-1}\left(\frac{C}{\omega }\right)$$

where 0 ≤ *θ*_1_ ≤ π/2 ≤ *θ*_2_ ≤ π, the two solutions of Equation (23); sin (*θ*_1_) = sin (π − *θ*_1_); *θ*_2_ = π − *θ*_1_. As the argument of the sine function of Equation (23) cannot be greater than 1 (*C*/*ω* ≤ 1), *C* equals the minimum angular velocity *ω*_min_. If *θ*_0_ ≤ π/2, then *ω*_0_ is increasing as *θ*_1_ is decreasing. If π/2 < *θ*_0_ ≤ π, then *ω*_0_ decreases first until it reaches *ω*_min_ at *θ* = π/2, followed by increasing *ω*. However, *θ* only decreases from *θ*_0_, first as *θ*_2_ and from *θ* = π/2 as *θ*_1_. In a numerical context, this condition is only satisfied when obeying the rule that d*θ*/d*t* < 0; i.e., calculating both *θ*_1_ and *θ*_2_ and select only consecutive *θ*-pairs whose d*θ*/d*t* is negative.

Substituting Equation (23) into Equation (14) yields

(24) $$-d\omega^{}\;I=dt^{}\;T_{R}\cos\theta =dt^{}\;T_{R}\cos\left[\sin^{-1}\left(\frac{C}{\omega }\right)\right]$$

Since $\cos\left[\sin^{-1}\left(C/x\right)\right]=\pm \sqrt{1-C^{2}/x^{2}}$, Equation (24) transforms to

(25) $$-d\omega^{}\;I=\pm dt^{}\;T_{R}\sqrt{1-\frac{C^{2}}{\omega^{2}}}$$

and

(26) $$dt=\pm \frac{I}{T_{R}}{\left(1-\frac{C^{2}}{\omega^{2}}\right)}^{-0.5}d\omega$$

Integrating both sides of the ordinary differential Equation (26) by considering *t*_0_ = 0

(27) $$t=\pm \frac{I}{T_{R}}\left(\sqrt{\omega^{2}-C^{2}}-\sqrt{\omega_{0}{}^{2}-C^{2}}\right)$$

Solving for *ω* as a function of time *t* yields:

(28) $$\omega =\sqrt{{\left(t^{}T_{R}/I\pm \sqrt{\omega_{0}{}^{2}-C^{2}}\right)}^{2}+C^{2}}$$

Equation (29) is only applicable if *T_R_* has a constant value. If *θ*_0_ = 0 then *C* = 0 and *ω* = *t T_R_*/*I* + *ω*_0_ = *t α* + *ω*_0_. The ‘±’ sign in front of the square root takes care of different *θ*_0_-conditions (not *θ*!): positive sign if *θ*_0_ < π/2 (Figure 3a), negative sign if *θ*_0_ > π/2 (Figure 3b). Equation (28) can be rewritten as:

(29) $$\omega =\sqrt{{\left(t^{}T_{R}/I+\mathrm{sgn}(\cos\theta_{0})\sqrt{\omega_{0}{}^{2}-C^{2}}\right)}^{2}+C^{2}}$$

which returns the correct *ω* at any *θ*_0_.

In summary, Equation (28) provides the analytic solution of *ω* as a function of time *t* (if *T_R_* is constant); and Equation (23) provides the analytic solutions of *θ*, as a function of *ω* but independent of *T_R_* (be it constant or a function of time).

The seemingly incredible relation of *θ* and *ω* in the absence of any influence by *T_R_*, as described by Equations (20)–(22), defines the unique dynamics of precession. It is explained from the left side of Equations (15) and (16), namely by the term d*t*·*T_R_*/*I*: if *T_R_* increases, then d*t* decreases and any instantaneous pair of *ω* and sin(*θ*), whose product is constant at all times, simply happens earlier and faster, i.e., at smaller times.

(h) Power *P*,

(30) $$P=\omega^{}T_{R}$$

(i) The energy problem of the precession: it is evident that energy is required to perform the precession of the spin axis. Precession occurs only if *θ*_0_ > 0. At the same magnitude of **T_R_**, the difference between *θ*_0_ = 0 and *θ*_0_ > 0 is that, in the former case, all energy produced by **T_R_** changes the magnitude of **ω**; whereas in the latter case, a certain percentage of this energy is wasted for precession. If *θ*_0_ = 90°, then at this moment, the percentage is 100%, as *T_R_* = *T_p_*, and *T_s_* = 0. In general, the angular kinetic energy *E_kin_* is calculated from:

(31) $$E_{kin}={\int P}^{}dt=I\frac{\omega^{2}}{2}$$

The energy problem of precession is subsequently explained by a simple example. A sphere of *I* = 1 m·kg^2^ is spinning at *ω*_0_ = 1 rad/s. A constant torque *T_R_* of 1 Nm is imparted on the sphere with a rectangular impulse of a duration of 1 s, whereby the initial angle *θ*_0_ between **ω_0_** and **T_R_** is 1.12 rad (64.16°; sin*θ*_0_ = 0.9). The initial angular kinetic energy *E*_0_ of the sphere before imparting the torque is 0.5 J from Equation (31). At *t* = 1 s after the *T_R_* was imparted, from Equations (28,29), *ω* = 1.695 rad/s. The actual *E_kin_* is therefore 1.436 J. Let us assume the ideal condition that *θ*_0_ = 0, i.e., all energy that can be potentially produced by *T_R_* accelerating *ω*. Then, from Equations (28,29), *ω* = 2 rad/s. The ideal *E_kin_* is therefore 2 J. Subtracting *E*_0_ from the two *E_kin_* provides the energies supplied by the torque, for actual and ideal conditions: *E_actual_* = 0.936 J, and *E_ideal_* = 1.5 J. Both *E_actual_* and *E_ideal_* represent a **change** (Δ) in energy, produced by the torque imparted on the sphere.

(j) The efficiency *η* is the ratio of actual energy to ideal energy:

(32) $$\eta =100\frac{E_{actual}}{E_{ideal}}$$

expressed as a percentage.

In the example above, this energy ratio equals to 0.936 J / 1.5 J = 0.624 and thus *η* = 62.4% at *t* = 1 s. The two energies, *E_actual_* and *E_ideal_* were calculated from two angular velocities (more precise: from changes in velocity squared), namely *ω_actual_*, identical to *ω_R_* (the vector sum of *ω_xyz_*) of Equation (2), and *ω_ideal_*,

(33) $$\omega_{ideal}=\int_{t_{1}}^{t_{2}}\alpha_{R}dt=\frac{1}{I}\int_{t_{1}}^{t_{2}}T_{R}dt$$

Equation (33) implies that (ideally, for maximum energy generation) the following three vectors, **ω_ideal_**, **α_R_**, and **T_R_**, are stationary and collinear, and that the angle *θ* between **ω_ideal_** and **T_R_** is 0.

The problem when calculating the angular kinetic energy is that it comes from two different sources, namely from the rotation of the bowling arm, serving to increase the translational (!) kinetic energy of the ball, and from the combined motion of forearm joints, wrist joints and finger joints, that generates the spin rate and the angular kinetic energy. Therefore, the change of energy Δ*E* has to be calculated, i.e., the differential of angular kinetic energy existing before imparting the torque spike on the ball (initial spin rate *ω*_0_ and its energy *E*_0_) and the terminal kinetic energy at release of the ball (terminal spin rate *ω_t_* and its energy, *E_t_*):

(34) $$\Delta E_{kin}=E_{t}-E_{0}=I\left(\frac{\omega_{t}{}^{2}}{2}-\frac{\omega_{0}{}^{2}}{2}\right)=0.5^{}\;I^{}\;\Delta \left(\omega^{2}\right)$$

Equation (32) is then updated to

(35) $$\eta =100\frac{\Delta \left(\omega_{actual}{}^{2}\right)}{\Delta \left(\omega_{ideal}{}^{2}\right)}$$

Using Δ*ω*^2^ avoids any influence of energy produced by a source other than the actual mechanism that is responsible for generating the spin rate of the ball immediately before release. This issue is also seen from the position of the spin axis when rotating the bowling arm and when imparting spin on the ball. This position changes from one action to the other, and thereby causes precession of the spin axis. This is most impressively seen in fast bowling, where the rotation of the arm produces a top spin of the ball, whereas the ball is released with a backspin. Consequently, the spin axis has to move from one side of the ball to the other [15,16], and as a result, the ball is temporarily decelerated (cf. Figure 3b), followed by an acceleration.

*ω*_0_ was determined at the time stamp *t*_1_, implemented in Equation (33), when *T_R_* exceeds 0.02 Nm. This threshold was determined empirically as the beginning of the torque spike. *ω_t_* was determined at the time stamp *t*_2_ after the *T_R_*-spike when the ratio *T_p_*/*T_R_* exceeds 0.98 (before reaching its maximum at 1). At this point, the aerodynamic torques are greater than 98% of the total torque *T_R_* and have almost entirely taken over the torques acting on the ball, whereas the finger torques (*T_s_*) dropped to negligible values. The fact that the release point of a spinning object happens when *T_p_*/*T_R_* reaches 1 was already suspected earlier but ultimately verified with a smart frisbee [30]. As a frisbee is released faster than a cricket ball, the transition from sub-unity *T_p_*/*T_R_* to unity *T_p_*/*T_R_* happens sharper and more suddenly than in a cricket ball which is characterised by a more gradual transition.

(i) ratio of peak angular acceleration before release to maximal spin rate at release, *α*_max_/*ω*_max_, has the unit s^–1^ and therefore corresponds to frequency. If *ω* were half a sine wave

(36) ω=asin(2πft)

where *a* is the amplitude and *f* is the frequency of the sine wave, then

(37) α=acos(2πft)2πf

Considering that the *ω*- and *α*-peaks occur at different times, e.g., *α*_max_ at *t* = 0 and *ω*_max_ at *t* = π/2, then

(38) $$\frac{\alpha_{\max}}{\omega_{\max}}=2\pi f$$

Thus, the frequency is

(39) $$f=\frac{\alpha_{\max}}{\omega_{\max}}/2\pi$$

and the cycle time is

(40) $$t=\frac{1}{f}=\frac{2\pi }{\alpha_{\max}/\omega_{\max}}$$

The half cycle time is then

(41) $$\frac{t}{2}=\frac{\pi }{\alpha_{\max}/\omega_{\max}}$$

The smaller the *α*_max_/*ω*_max_ ratio, the longer is the cycle period, and the more spin rate is produced relative to the angular acceleration.

The performance parameters described so far are divided into physical performance and skill performance as shown in Table 1. Although the physical performance parameters are primarily generated by the muscle force, they are also influenced by the skill parameters.

Although the five different skill parameters are related to each other (as shown in the Results section), they reflect different aspects of the generation of spin rate: *p_n_* (*θ*_0_), *p*, and *T_p_* are peak values that do not occur at the same time, but rather in the order as indicated, all of them before the *T_R_*-peak (Figure 5). All performance parameters, physical and skill, grant a unique and detailed view into the biomechanics of how the spin rate of the ball is generated.

### 2.3. Applications of the Performance Parameters

We conducted four different studies to scope the applications of the performance parameters obtained from the smart ball.

#### 2.3.1. Study 1

This study served to understand how the skill parameters are related to each other, and how they can be used for TID (talent identification). 33 bowlers participated in this study, 7 wrist spinners, 16 finger spinners, and 10 fast bowlers (seam bowling), ranging from amateur level to national team players. Note that the fast bowlers were included because fast bowling deliveries are expected to be very inefficient, as already mentioned in the Performance Parameter section above, and therefore serve as a means for comparison. The number of participants in each cohort is justified from a recent and comparable study where physical performance parameters were used for profiling 11 bowlers and 8 batsmen [31]. All 33 participants bowled the ball at least six times. We analysed only comparable deliveries within each group, namely sidespin (leg-spin) in wrist spinners, sidespin (off-spin) in finger spinners, and seam bowling in fast bowlers. We processed the results of different performance and skill parameters per bowler with multiple regression analysis and determined the R^2^ value to identify by how much an individual skill parameter is uninfluenced by the remaining four.

#### 2.3.2. Study 2

This study served to understand the influence of different deliveries on the skill parameters, thereby reflecting the bowling ‘efficiency’ of different deliveries. One author of this publication (REDF) served as a participant in this study, by bowling twelve times each of the seven different finger-spin deliveries (Flipper, backspin, back-sidespin, sidespin, top-sidespin, topspin, Doosra) and each of the six wrist-spin deliveries (backspin, back-sidespin, sidespin, top-sidespin, topspin, Googly). The participant was a first-class cricketer and is capable of bowling the different spin bowling deliveries at similar performance levels. This explains why only one bowler was tested, as the emphasis of this study was on the understanding of the differences between the deliveries. The accuracy of the deliveries was verified by classifying the deliveries with a 14-camera Cortex Motion Analysis system (vers. 3.1 Motion Analysis Corp., USA) operating at 200 Hz, by attaching 3 markers to the surface of the ball, 50–65 mm apart. The three markers were used to calculate a local orthogonal coordinate system for the ball using the skeleton builder function of the Kintools RT software (Version 2, Motion Analysis Corporation Ltd.), which performs kinetics calculations within the Cortex software. From the global coordinates of the three markers, we calculated the helical axis of the ball rotating during flight (spin axis, angular velocity vector) as well as the ball’s centre of mass and its translational velocity vector. We used the direction of the spin axis vector relative to the velocity vector of the ball as a criterion for the classification of the deliveries.

#### 2.3.3. Study 3

This exploratory study served to understand the dynamics of the performance parameters (physical and skill) during a 10-over spell. We did not expect conclusive results in the first place but prioritised the development of a protocol to explore how 10-over spell data can be interpreted for performance analysis, specifically for profiling, talent identification and potential for improvement. For this purpose, only three bowlers participated in this study, whose stock balls were finger-spin topsidespin and wrist-spin sidespin. The three participants bowled the smart ball 60 times (10 overs) consecutively. We assessed the performance parameters against the time (number of the delivery) in terms of the R^2^ value of the regression slope and its p-value, as well as in terms of the residual standard deviation and the differential of the predicted values of first and last balls, to identify any improvement (training effect) or decline (e.g., due to fatigue) of the performance parameters.

#### 2.3.4. Study 4

This study served to exemplify the usefulness of the smart ball for performance analysis and for monitoring the success of training intervention. One bowler, whose stock ball was the finger-spin top-sidespin, underwent a routine performance test. Study 4 is a unique case report, and this is why only one bowler was included. After identification of a bowling technique problem from the performance data, the coach (REDF) corrected the technique with training intervention. Subsequently, the performance was re-assessed with the smart cricket ball. Furthermore, the coach bowled both the correct and the incorrect technique for comparative reasons. The changes in performance before and after intervention training were assessed with the Mann–Whitney U test and evaluated from the p-value and the effect size r (r = 1 − 2U/Π(n); interpretation according to McGrath and Meyer [32]).

To distinguish between finger-spin and wrist-spin deliveries independent of the handedness of the bowler, the ball’s coordinate system was aligned in the following way: the positive z-axis (perpendicular to the seam plane) pointed to the right in the global coordinate system (i.e., for spin bowlers out of the palm in lefthanders, and into the palm in righthanders), and the tip of the index finger was aligned to the positive x-axis (whereby the index was placed across the seam in spin bowling, and at the side of the seam in seam bowling). In finger-spin deliveries, the sense of **ω** was negative, whereas the sense of **ω** was positive in wrist-spin deliveries (but also in fast bowling). Therefore, the flipper delivery was identified as a finger-spin delivery (cf. Study 2 above), in contrast to the common, but incorrect, belief it be a wrist-spin delivery (cf. figure 5.19 of [2]).

This research was granted Ethics approval by the RMIT University Human Ethics Committee (approval no. BSEHAPP 13-12), and the Swinburne University Human Ethics Committee (approval no. 20191582-3216) and adhered to the Declaration of Helsinki.

## 3. Results

### 3.1. Study 1

**Figure 6:**
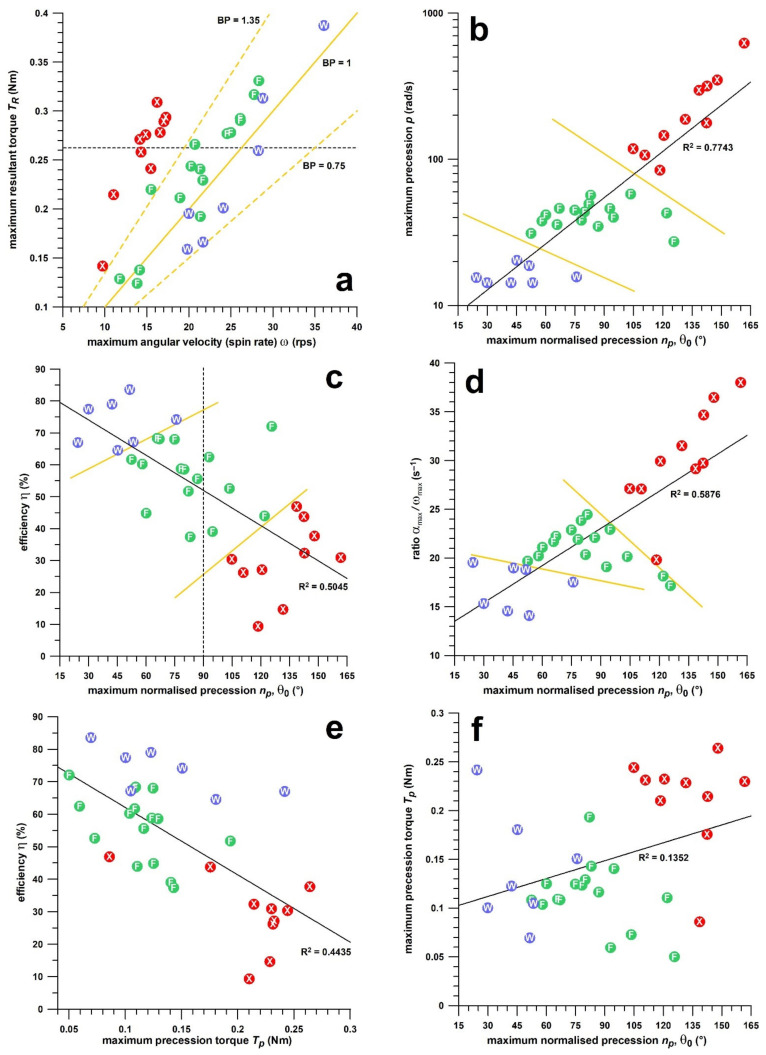
Correlation of different performance parameters; red dots (white X): fast bowlers; green dots (white F): finger-spinners; blue dots (white W): wrist-spinners; (**a**) torque vs spin rate, the yellow isolines indicate the bowling potential BP at three different levels; the dashed horizontal isoline indicates a torque of 0.26–0.265 Nm, corresponding to 14 rps spin rate in fast bowlers, 21 rps in finger-spinners, and 28 rps in wrist-spinners; note that the wrist-spinners generate twice the spin rate than the fast bowlers at this torque level (*this subfigure utilises the data shown in figure 1 of Fuss et al.* [22]*, © 2020 by the authors. Licensee MDPI, Basel, Switzerland*.); (**b**) precession vs normalised precession; the two yellow lines separate the three bowler cohorts; (**c**) efficiency vs normalised precession; the two yellow lines separate the three bowler cohorts; the dashed vertical line at 90° separates the bowlers with *θ*_0_ (initial the angle between the spin rate vector **ω** and the torque vector **T_R_**) smaller (Figure 3a) and greater (Figure 3b) than 90°; (**d**) *α*_max_/*ω*_max_ vs. normalised precession; the two yellow lines separate the three bowler cohorts; (**e**) efficiency vs normalised precession; (**f**) precession torque vs normalised precession.

The importance of skill parameters is exemplified in Figure 6a where a resultant torque *T_R_* of 0.26–0.265 Nm produces a spin rate *ω* of 14 rps in a fast bowler (X in Figure 6a), 21 rps in a finger spinner (F in Figure 6a), and 28 rps in a wrist spinner (W in Figure 6a). The wrist spinner generates two times more spin rate than the fast bowler—but at the same torque expenditure. This striking result is not only predominantly due to the three different deliveries, but also due to the skill of the bowler. When dividing the torque *T_R_* (Nm) by *ω* (rps), we obtain the bowling potential (BP in Figure 6a), with the rule of the thumb that 0.1 Nm produces a spin rate of 10 rps (BP = 1; [22]). In Figure 6a, the BP in spin bowlers ranges from 0.75 to more than 1.35. The larger the BP, the more potential the bowlers have to improve their skills by reducing, for example, the precession, which had prevented them from producing more energy in the first place. At a small BP, the skill has already reached a high level of proficiency, and the only practical way for improving the spin rate is through muscle training.

As already mentioned in the Performance Parameters section, the skill parameters reflect different aspects of the generation of the spin rate. Table 2 shows how the magnitude of an individual skill parameter is explained or rather not explained from the magnitudes of the remaining four skill parameters. While the decadic logarithm of the precession or the ratio *α*_max_/*ω*_max_ does not depend on the other four skill parameters in less than 7% for all data (spin and seam bowling combined, as well as seam bowling alone), this percentage ranges from 15 to 49% in spin bowling for all five skill parameters.

Figure 6b–f shows the correlation of selected skill parameters and how they influence each other. While the correlation of *p* and *p_n_* has a high R^2^ value and clearly separates the cohorts of the three different deliveries, the smaller the R^2^ value becomes, the more the three cohorts merge and overlap. In Figure 6b, while the wrist-spin’s precession ranges only from 14 to 16 rad/s, the data range of the normalised precession spans from 24° to 76°. The opposite is true for seam bowling, 84–241 rad/s versus 105°–132°. In short, within a single delivery, there is no evidence of a perfect correlation between two single skill performance parameters, because they represent the performance of different aspects of spin generation.

### 3.2. Study 2

**Figure 7:**
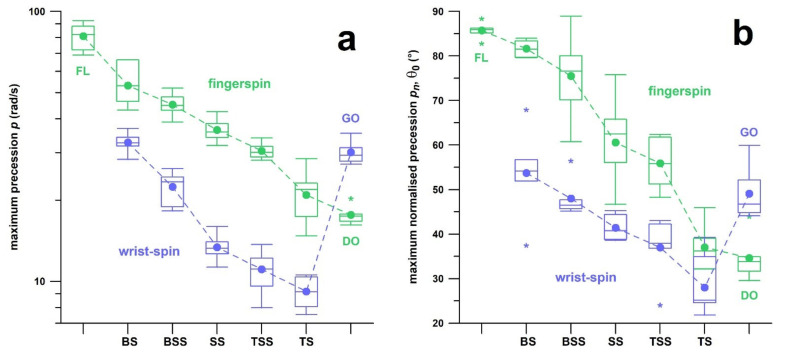

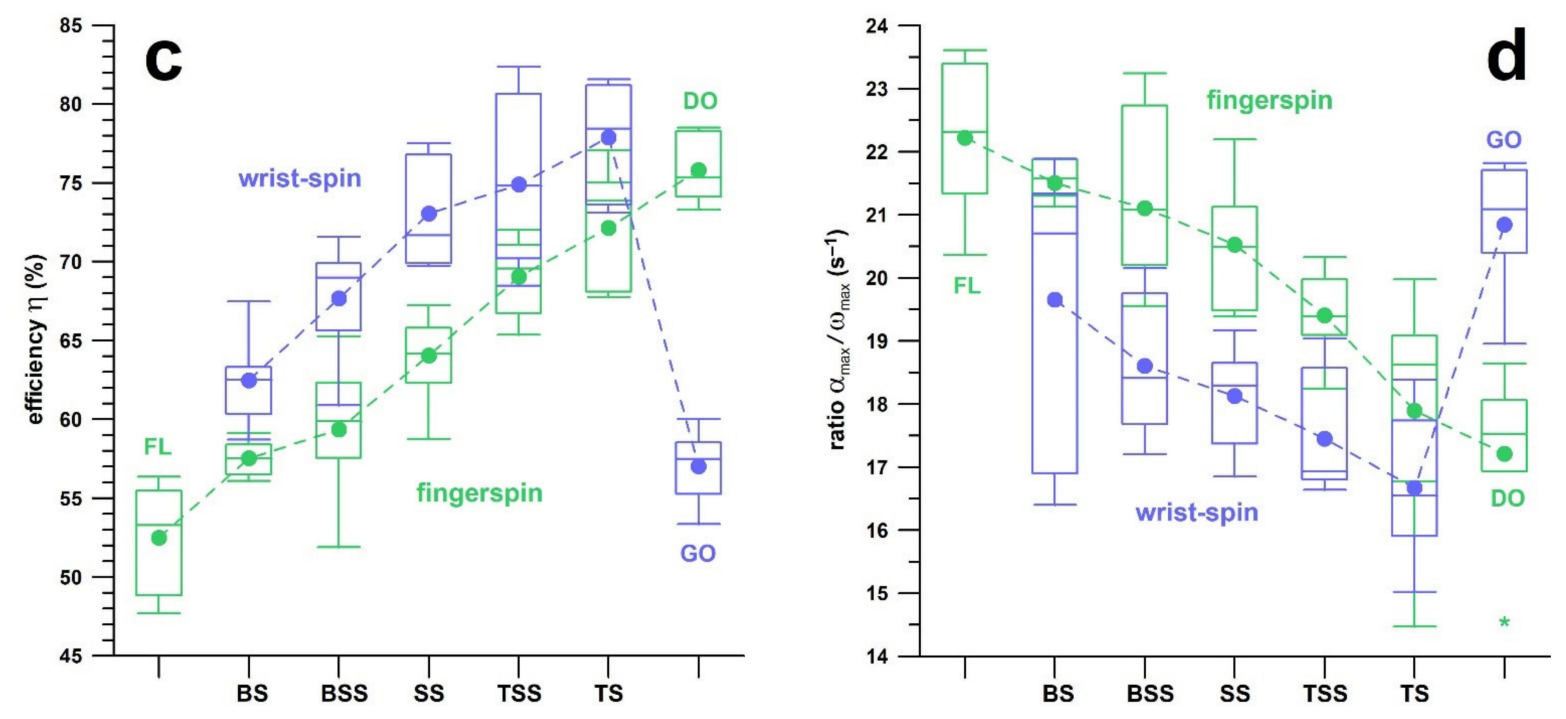
Skill performance parameters (box plots and averages) vs. type of delivery; the skill performance parameters are: (**a**) precession; (b) normalised precession; (**c**) efficiency; (**d**) ratio α/ω; green: finger-spin; blue: wrist-spin; BS = backspin, BSS = backsidespin, SS = sidespin, TSS = topsidespin, TS = topspin; the transition from BS to TS is continuous; FL = Flipper; DO = Doosra; GO = Googly; the asterisks denote outliers.

In contrast to the skill performance parameters of individual bowlers (within the same delivery; sidespin in study 1), different deliveries show a common trend across the skill performance parameters. This trend shown in Figure 7 reveals that wrist-spin deliveries are more efficient than corresponding finger-spin deliveries, and topspin deliveries are more efficient than backspin deliveries. Furthermore, the trend exhibits a continuous transition from backspin over sidespin to topspin. The only exception is the Googly: although it is related to the topspin delivery, its efficiency is comparable to, if not worse than the backspin delivery. Even if the Flipper and the Doosra are backspin and topspin deliveries, respectively, their efficiency is consistent, worse and better, respectively, than their nearest neighbours (backspin and topspin, respectively; Figure 7).

### 3.3. Study 3

**Figure 8:**
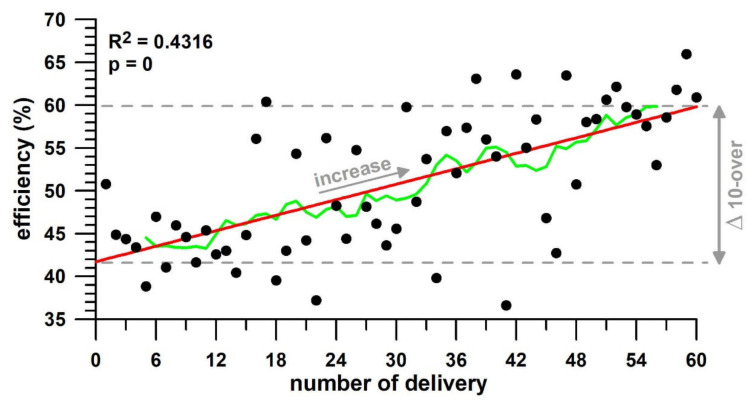
10-over spell of participant 3 (Table 3; best improvement of all performance parameters across all participants); efficiency vs. number of delivery (bowling of 6 balls = 1 over); linear fit (red) and running average (green, window width of 9 data).

Table 3 shows the statistical data of the 10-over spell tests. Figure 8 explains the statistical data of Table 3 graphically, exemplified by the parameter with the best improvement (efficiency of participant 3). In Table 3, the mean indicates the overall performance (skill and physical) of the participants. The RMSE (root-mean-square error; residual standard deviation) represents the fluctuations (Figure 8) of the data about the linear regression fit line; and the RMSE% is the RMSE normalised to the mean. The *R^2^* value explains by how much the change of a parameter can be explained from how often the ball is bowled. The *p*-value informs us of the likelihood that the trend observed is due to chance (if *p* > 0.1 then the likelihood is >10%; alpha = 0.1). The Δ *10-over* value provides the change of the dependent parameter, across the spell, estimated from the model (if *p* < 0.1).

Participant 1 (stock ball: finger-spin topsidespin) shows gains in *physical* performance for the following parameters: spin rate *ω* (marginally significant), torques *T_R_* and *T_s_* (marginally significant), and power *P* (marginally significant). The effect on power is explained from *ω* and *T_s_* (i.e., from their product). Participant 1 shows losses in *skill* performance, namely with the normalised precession *p_n_* and the efficiency *η*. Realistically, irrespective of any p-value considerations, the authors of this paper consider an increase in *p_n_* of 7° as substantial from experience; whereas, all other changes, particularly the increase in *T_R_* and the decrease of *η* are considered trivial.

Participant 2 (stock ball: wrist-spin sidespin) shows gains of performance in *ω* (marginally significant), *T_R_* and *α*/*ω*, while a loss in *T_s_*. The effect on *α*/*ω* is explained from the gain in *ω* and the loss in *T_s_*, considering that *α* = *T_s_*/I. The gain in *α*/*ω* is substantial, the trend effect on *T_R_* and *T_s_* is medium, and the effect on *ω* is low.

Participant 3 (stock ball: finger-spin topsidespin) shows gains of performance in *α*/*ω* and *η*; while losses in *ω*, *T_R_* (marginally significant), *T_s_*, and *P*. (which is the opposite effect seen in participant 1, except for *α*/*ω*, not affected in participant 1). The effect on *η* is considered substantial (very large); on *P* large; on *T_s_* medium, and on *ω* low.

Comparing the 3 participants among each other, as a profiling exercise, participant 2 shows the fastest spin rate, on average; whereas, participant 1 generates the greatest torque (*T_R_* and *T_s_*) and power. These two profiles were different because participant 2 bowled wrist-spin and therefore could generate more spin from a smaller torque. A striking result is the torque distribution of *T_R_*:*T_s_*:*T_p_* = 0.28:0.22:0.20 of participant 2 with a relatively high *T_p_*, in contrast to participant 1 (0.29:0.28:0.11) and participant 3 (0.24:0.22:0.12). This result corresponds to a lower level of skill performance of participant 2. Accordingly, we would be inclined to assume that the remaining four skill parameters of participant 2 correspond to lower skill levels too. However, since participant 2 is a wrist-spinner, it is not surprising that he produces the lowest precession *p*, the 2nd lowest *p_n_*, the highest *η*, and the lowest *α*/*ω*. This result emphasises the importance of considering a bowler’s performance relative to the type of delivery (Figure 7) to prevent misinterpretations. Furthermore, this result, although extreme, mirrors the data shown in Table 2, namely that approximately 50% of *T*_p_ cannot be explained from the other four performance parameters. It has to be emphasised at this point that we are comparing peak values here.

**Figure 9:**
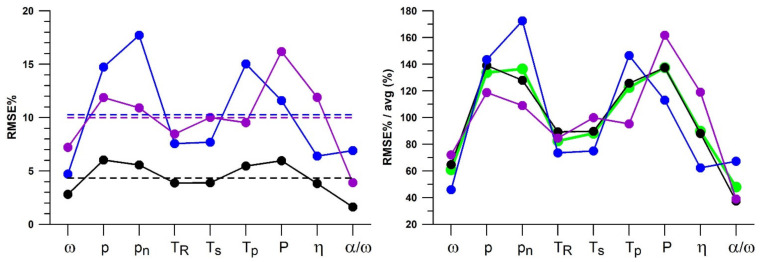
RMSE% and RMSE% normalised to the average (avg) RMSE% of each participant (black: participant 1; blue: participant 2; purple; participant 3) and each performance parameter shown in Table 3 (residual standard deviations during the 10-over spells); the dashed lines show the average RMSE% of each participant, used for normalising the RMSE% in the right graph; the green line corresponds to the average of all participants.

In addition to the magnitude of the nine different parameters (mean in Table 3), a further aspect of the 10-over spell test is the fluctuation of the data across the regression fit (Figure 8), corresponding to the RMSE (residual standard deviation) and RMSE% data in Table 3, shown graphically in Figure 9. Participant 1, who is the most experienced of the three, has the least RMSE% on average. There is a trend across the performance parameters, namely that *p* and *p_n_*, as well as *T_p_* and *P* exhibit a higher RMSE% than the other parameters.

### 3.4. Study 4

**Figure 10:**
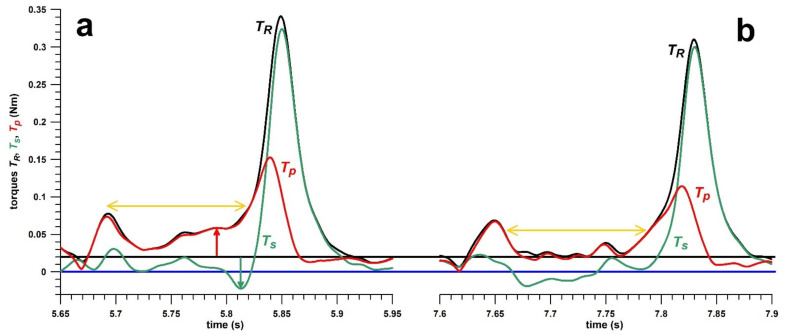
Torques vs. time before (**a**) and after (**b**) intervention training; *T_R_* = resultant torque (black); *T_s_* = spin torque (green), *T_p_* = precession torque (red); the red and green arrows in subfigure (**a**) indicate the increased *T_p_* and negative *T_s_*; the blue horizontal line indicates the baseline at 0 Nm, black horizontal line indicates the threshold at 0.02 Nm (cf. main text); the yellow double arrow defines the 0.123 s window for calculating the average *T_p_* (ending at last negative *T_s_* peak before the main *T_R_* peak).

In this case study, a promising bowler with a stock ball of finger-spin topsidespin was tested for physical and skill performance. After bowling the first ball and immediately processing the data (Figure 10a) it became clear that the precession torque *T_p_* was unusually high (red arrow in Figure 10a), and the spin torque *T_s_* was even negative (green arrow in Figure 10a) immediately before the main spike. *T_s_* is negative only if *θ*_0_ (*p_n_*) is greater than 90°, typical for fast bowlers (Figure 6b–d,f). As expected, the average *θ*_0_ was 94.69°. The statistics of the profiling exercise are shown in Table 4.

**Figure 11:**
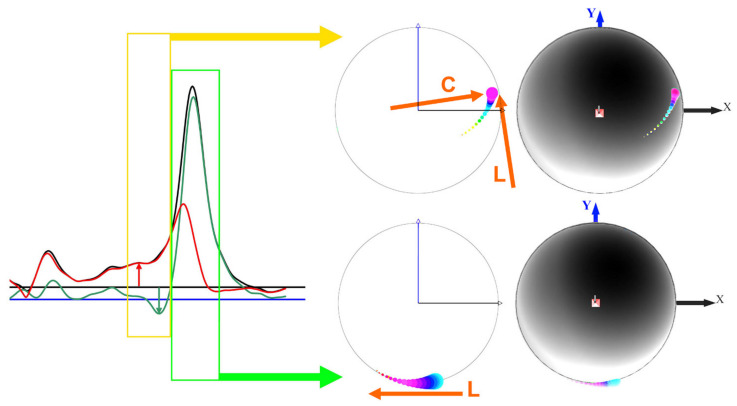
Different sections of the torque vs time graph of Figure 10a, used for calculating the moving centre of pressure (COP); L = COP movement along the seam; C = COP movement across the seam; X and Y: smart ball coordinate system.

To investigate this issue, the diagnostic process started with assessing the path of the centre of pressure (COP; [19]). The COP at the torque peak (Figure 11, green box) moved along the seam as expected from spin bowlers [19]. However, when concentrating on the time window (Figure 11, yellow box) where the problem was detected, the COP moved halfway across and along the seam. The COP moves along the seam if the spin axis is approximately perpendicular to the plane of the seam, typical for spin bowling; and across the seam if the spin axis is aligned parallel to the plane of the seam. This is only possible if the ball is rotated about the axis of the forearm (pronation/supination). A closer look at the bowling movement revealed that the bowler, although bowling a topspin (finger-spin topsidespin, with the forearm halfway between pronation and supination), had the forearm unusually rotated for a backspin delivery (hyper-pronation) and then supinated the arm rapidly to release the ball with a topsidespin.

**Figure 12:**
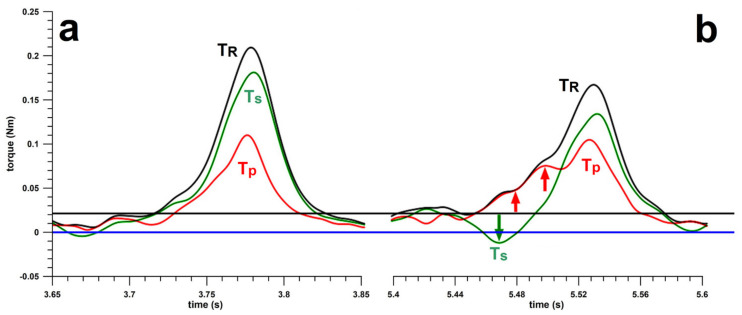
Data of the coach bowling a standard topsidespin (**a**) in the traditional way and re-enacting the bowler’s technique (**b**) shown in Figure 10a; T_R_ = resultant torque (black); T_s_ = spin torque (green), T_p_ = precession torque (red); the red and green arrows in subfigure (**b**) indicate the increased T_p_ and negative T_s_; the blue horizontal line indicates the baseline at 0 Nm, black horizontal line indicates the threshold at 0.02 Nm (cf. main text).

To verify the torque data shown in Figure 10a, the coach (REDF) bowled the finger-spin topsidespin delivery in the usual way (Figure 12a) and in the unusual one (with a backspin approach; Figure 12b), revealing the same pattern (green and red arrows in Figure 12b). The major difference in the coach’s data was that *T_R_* and *T_s_* (and thus *ω*) were smaller when bowling the unusual technique, which was not surprising as the coach was not used to the new unusual bowling movement. The magnitude of the peak *T_p_* was not smaller when bowling the unusual technique, however, when normalising it to the *T_R_*, it was comparatively higher in the unusual technique. The difference in efficiency *η* was 73% for the traditional technique, and 37% for the unusual one.

The bowler underwent a 3-h intervention training session during which the coach retrained the bowler to the traditional technique, followed by a further profiling assessment. The torque data (Figure 10b) changed substantially, as seen from Table 4, particularly the magnitude of the downward *T_s_* spike became positive, and the average *T_p_* over a 0.123 s window (Figure 10) decreased significantly (*T_s_***___**_min_: p = 0.0006, effect size r = 0.83/large effect; *T_p_***___**_avg_: *p* < 0.0001, effect size r = 1/large). The physical performance parameters decreased significantly (*ω*, *T_R_*, *T_s_*, *α*, *P*; all of them at *p* < 0.0001, and effect size r = 1/large), as the bowler was not used to the traditional technique (the same effect as seen in the coach’s data; Figure 12). However, in terms of skill performance parameters, efficiency *η*, normalised precession *p_n_*, precession torque *T_p_* and the ratio *α*_max_/*ω*_max_ improved significantly (*η* from 39% to 59% [*p* < 0.0001, effect size r = 1/large]; *p_n_* from 95° to 78° [p = 0.0006, effect size r = 0.83/large]; *T_p_* from 0.1405 to 0.1234 Nm [p = 0.0226, r = 0.56/large); *α*_max_/*ω*_max_ from 22.93 to 21.91 s^–1^ [*p* < 0.0001, r = 1/large]).

**Figure 13:**
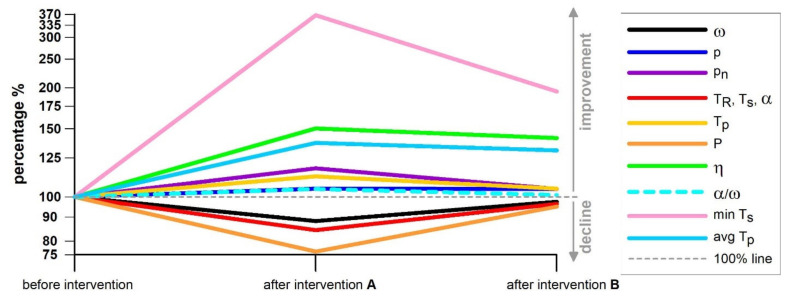
Data of Table 4 in graphical form; the data before the training intervention are set to 100%, and the data after the intervention are shown relative to the data before the intervention; percentage > 100% = improvement of a skill parameter after intervention; percentage < 100% = decline of all 5 physical performance parameters after intervention; after intervention (**A**) = immediately after the intervention; after intervention (**B**) = after several days of self-training; min T_s_ and avg T_p_ refer to the yellow double arrow in Figure 10, defining the 0.123 s window for calculating the average T_p_, ending at last negative T_s_ peak before the main T_R_ peak.

After several days of self-training, the bowler returned for another profiling test. The physical performance parameters improved through the training effect and achieved values almost as high as before the intervention: *ω* and *T_R_*: p = 0.0688, effect size r = 0.44/large; *T_s_* and *α*: p = 0.0226, effect size r = 0.56/large; *P*: p = 0.0404, r = 0.5/large); *p_n_* worsened to 90° (not different from *p_n_* before the intervention training), *η* dropped only slightly to 55% (*p* < 0.0001, effect size r = 1/large); *T_s_min_* decreased but was still far better than the data before the intervention training, whereas *T_p_*__avg_ worsened slightly but was still better than the average before the intervention training (*p* < 0.0001, effect size r = 1/large). The data in Table 4 are summarised graphically in Figure 13.

## 4. Discussion

The key to bowling skill (at least for spin bowling) is the peak normalised precession *p_n_*, identical to *θ*_0_, the angle between **ω** and **T_R_**. The closer **ω** and **T_R_** are, the smaller is *θ*_0_, and the more torque is converted to *ω* and to angular kinetic energy, rather than wasted for precession. *θ*_0_ depends on how fluently the movements are executed, but also on the type of the delivery. A striking example of additional but unnecessary movements of a baseball pitcher was given by Doljin et al. [33]. The pitching efficiency of this player was only 5.4%, the spin rate a low 15.7 rps, but the torque was extraordinarily high at 0.396 Nm. A wrist spinner could have easily achieved 40 rps with the same torque.

In the case of profiling, a spin bowler should be tested for at least 2 overs (12 balls bowled) and the physical and skill performance parameters should be analysed. Selecting a spin bowler based solely on spin rate shows only the confounded effect of physical and skill performance but does not reveal any causal mechanisms. The cause of high or low spin rate is not only the magnitude of the torque *T_R_* but also, even more important, how efficiently the torque is imparted for the purpose of generating the spin rate *ω*, gauged from the skill parameters. The first step for doing this is consulting the bowling potential (BP, Figure 6a), i.e., 100 *T_R_* (Nm)/*ω* (rps). The greater it is, the less skilful are the bowlers, but the more potential they have of increasing their spin rate when improving their lacking skill. In other words, the greater the BP, the more scope the bowlers have to improve their spin rate by becoming more technically proficient through training.

The comparison of different spin bowling deliveries provides a means of assessing bowling performance that exceeds the practice of merely measuring spin rate and other simple performance outcomes. Any profiling exercise has to start with classifying the stock ball of the spin bowler, as well as testing other deliveries a bowler can master. This can be done with a motion analysis system [18], or directly with the smart ball (Fuss et al., unpublished data). The data shown in Figure 7 can be used as a guideline of a high skill performance. Yet, while the *p_n_* data of the wrist-spin sidespin delivery shown in Figure 7b are 41° on average, the wrist-spinner with the fastest spin rate (36 rps) shown in Figure 6a had a normalised precession *p_n_* of 24°, the smallest of all data shown in Figure 6b.

Burnett et al. [34] investigated the effect of a 12-over bowling spell on kinematic parameters of fast bowlers and concluded that the “*fast bowling technique does not change over this length of spell*”. Schaefer et al. [35] conducted a similar study on the effect of a 10-over spell on kinematic and kinetic parameters of junior fast bowlers, and did not find substantial changes “*in mean values or variability of any kinematic, kinetic or performance variables*”. In contrast to these two literature sources, we could detect significant trends for both physical and skill parameters in terms of both losses and gains in performance (Table 3) in a 10-over spell, but these trends are different from bowler to bowler. For example, if the efficiency increases (as seen in participant 3; Table 3 and Figure 8) over the spell, then there is evidence of a training effect. Profiling of the three bowlers who participated in the 10-over spell test by merely considering their skill performance data would suggest that participant 2 has the best skill performance. However, participant 2 bowled a wrist spin in contrast to participants 1 and 3 who bowled a finger spin, which explains that the alleged high performance is just an effect of the type of delivery (Figure 7). Another method for performance assessment based on 10-over spell data seems to be the data fluctuation throughout the spell, expressed by means to the RMSE%. Participant 1 is a first-class cricketer exhibiting smaller fluctuations, whereas participants 2 and 3 are less experienced (Figure 9) with pronounced fluctuations. It remains to be tested whether a smaller RMSE% is related to less variability and to a higher experience of a bowler.

The case report of the training intervention shows the application of the smart ball for performance diagnostics and training monitoring. However, the question could be asked whether the correction of the technique was really necessary? The cricketer was able to bowl the ball perfectly well, despite lower efficiency and thus more potential to improve. Yet, the bowler is now able to perform the same delivery (topspin) with two different techniques, which can be mistaken easily for two different deliveries (backspin and topspin). This principle challenges the traditional view of spin bowling deliveries, considering that the traditional classification tree of spin bowling deliveries [2] has been extended and improved anyway in this paper, specifically in Figure 7, by proving a continuum from backspin to topspin deliveries. Equally, the Flipper was identified as a finger-spin delivery (formerly regarded as a wrist-spin delivery [2]); and the Doosra is equally a finger-spin delivery (formerly considered a hybrid of a finger- and wrist-spin [2]).

Batsmen are confounded by differences between the types of spin bowling deliveries to the degree in which their arm and hand kinematics are similar. For example, the wrist-spinner’s top spin has the same arm and hand orientation as the finger-spinner’s flipper (i.e., back spinner), making it harder for batsmen to discriminate between these two deliveries despite the opposite directions of applied spin torque by the fingers. An easier discrimination would be made between the finger-spinner’s top-spinner and the flipper since the orientation of the palm through ball release is opposite (i.e., medial versus lateral). The same principle of confusion arises from the two different techniques of bowling a topspin (Figure 10): the inefficient one (Figure 10a) which looks like a backspin, and the traditional one (Figure 10b) which looks like a topspin. The major difference between the standard source of confusion and the apparently new one (Figure 10), never described before, is that the former is characterised by the same arm and hand orientation despite opposite spin directions; and the latter is marked by the same spin direction despite different arm and hand orientation. In summary, inefficient bowling techniques (Figure 10a) could hinder potential improvement and lead to diminished performance over a spell of bowling, and that inefficient spin bowling variations can be chosen for the purposes of disguise—i.e., increase the difficulty of the batter to discriminate between delivery types. In other words, there is an interesting trade-off between bowling efficiency and disguise.

The limitation of our smart ball is that it does not measure the translational speed at the release of the ball, as we did not incorporate accelerometers into the ball. The reason for this was that we focussed on the spin rate, which is more important than the translational speed in spin bowling, and also enabled computing the ten performance parameters. Conversely, calculating the spin rate from the magnetometer signal [24], as done by commercially available smart balls (baseball and cricket), does not allow determining further parameters beyond the spin rate (at release or pre/post-bounce). Note that all 10 performance parameters (Table 1) are calculated merely from the variable angular velocity (and the moment of inertia of the ball which is just a constant). The reason for calculating the spin rate from the magnetometer signal is twofold: it saves space and is cheap, essential for commercialisation. Space is a relative term, as the size of the electronics components of our smart ball fits into a sphere of half the diameter of a cricket ball.

A further limitation of our study is the small sample size of Study 3, the 10-over spell test. As mentioned above, the studies of Burnett et al. [34] and Schaefer et al. [35] have not been able to detect kinematic changes over bowling spells. However, the smart ball can detect statistically significant changes to skill parameters that were previously unknown in cricket biomechanics. Our research was of an exploratory nature, developed to detect fatigue and training effects over a spell of bowling, both of which are essential for performance profiling. To date, a spell of several overs is only used for training purposes but not for performance analysis based on measured or calculated data. As such, the analysis introduced in the Results section (Table 3; Figure 8 and Figure 9) will serve as a template or standard for future studies. Specifically, there is evidence for distinguishing between training (gain of performance) and fatigue effects (loss of performance) as well as classification of performance based on the RMSE%. After formulating the hypotheses from the existing evidence, testing these hypotheses will open another completely new research area in the field of cricket.

## 5. Conclusions

Our smart cricket ball is a classic example of mobile computing for sports performance analysis. This analysis can be conducted indoors as well as outdoors, e.g., at the oval. The data are transmitted wirelessly after each delivery and processed instantly by the software so that the 10 performance parameters are readily available. The high data sampling frequency (815 Hz) and the three high-speed (uniaxial) gyros are essential for precise computing of the five physical and five skill performance parameters.

Commercially available smart balls provide only a single datum of the spin rate at release, without providing any reason why the spin rate is higher or lower in one bowler, compared to other bowlers. As explained in this paper, wristspin and topspin deliveries are more efficient than fingerspin and backspin ones. From a practical point of view, care should be taken when using only the spin rate as a performance parameter for profiling: two bowlers with the same physical and skill performance level cannot produce the same spin rate if one bowler is a fingerspin-topspinner and the other is a fingerspin-backspinner.

As of now, there is no competitive product to the high-speed gyroscope ADXRS649 (Analog Devices, Wilmington, MA, USA) because it is patented [29]. In the future, the high-speed gyros will become cheaper and smaller, so that a more thorough and detailed performance analysis opens up the scope for sophisticated talent identification and training. Spin bowling is considered a lost art. The smart cricket ball is designed to revive it.

As a side effect of this paper, we discovered a new mechanical principle never reported in a mechanics textbook so far, namely that the product of the angular velocity *ω* and the sine of the angle *θ* (between the torque and the angular velocity vectors) remains constant at any moment in time during the precession of the angular velocity vector.

## Figures and Tables

**Table 1:** Performance parameters obtained from the smart cricket ball.

Physical Performance Parameters	Training Target	Skill Performance Parameters	Training Target
Maximum spin rate *ω_R_*	Improve	Maximum precession *p* (before the torque spike)	Reduce
Maximum angular acceleration *α*	Improve	Maximum normalised precession *p_n_* = *θ* (before the torque spike)	Reduce
Maximum resultant torque *T_R_*	Improve	Maximum precession torque *T_p_*	Reduce
Maximum spin torque *T_s_*	Improve	Efficiency *η*	Improve
Maximum power *P*	Improve	‘Frequency’ *α*_max_/*ω*_max_	Reduce

**Table 2:** Unaccounted percentage of a specific skill parameter, not explained from the other four skill parameters, calculated from 100 minus 100 · R^2^ of a multiple regression; e.g., 4.25% means that the magnitude of the decadic logarithm of the precession (dependent parameter) does not depend on the remaining four (independent) skill parameters in 4.25%; F = finger-spinners, W = wrist-spinners, X = fast bowlers; log_10_*p*_max_ = decadic logarithm of the precession; *p_n__*_max_ = normalised precession; *T_p__*_max_ = precession torque; *η* = efficiency; *α*_max_/*ω*_max_ = ratio of maximal angular acceleration to maximum angular velocity (spin rate).

Skill Parameter	Log_10_*p*_max_	*p_n__*_max_	*T_p__*_max_	*η*	*α*_max_/*ω*_max_
F + W + X	4.25%	18.63%	32.29%	23.19%	6.66%
F + W	15.18%	43.69%	48.51%	34.69%	27.47%
X	5.41%	15.97%	25.89%	38.53%	6.77%

**Table 3:** Statistical data of physical and skill parameters during a 10-over spell; data in bold font: significant trend if *p* < alpha (alpha = 0.1); italicised items: marginally significant, 0.1 < *p* < 0.12; RMSE = root-mean-square error (deviation) = residual standard deviation; RMSE% (= CV_RMSD_) = 100 · RMSE/mean; Δ 10-over: differential between predicted values of 1st and last ball; ‘effect on performance’ according to Table 1; ‘%change’ = 100 · (Δ 10-over)/mean.

	Spin Rate *ω* (rps)	Maximum Precession *p* (rad/s)	Maximum Normalised Precession *p_n_* (deg)	Maximum Resultant Torque *T_R_* (Nm)	Maximum Spin Torque *T_s_* (Nm)	Maximum Precession Torque *T_p_* (Nm)	Maximum Power *P* (W)	Effici-ency *η* (%)	Ratio α_max_/ω_max_ (s^–1^)
10-over spell, participant 1 (finger-spin topsidespin)									
Mean	25.95	33.40	89.29	0.2913	0.2815	0.1142	26.83	54.76	22.20
RMSE	0.73	2.02	4.93	0.0113	0.0101	0.0062	1.60	2.10	0.36
RMSE% (= CV_RMSD_)	2.81	6.04	5.56	3.88	3.90	5.46	5.97	3.83	1.63
R^2^	0.0402	0.0146	0.1522	0.0466	0.0429	0.0293	0.0402	0.0559	0.0233
p-value (alpha = 0.1)	*0.1171*	0.3510	**0.0017**	**0.0924**	*0.1062*	0.1821	*0.1194*	**0.0650**	0.2349
Trend of regression	*Increase*	nil	**Increase**	**Increase**	*Increase*	nil	*Increase*	**Decrease**	nil
Effect on performance	*Gain*	nil	**Loss**	**Gain**	*Gain*	nil	*Gain*	**Loss**	nil
Δ 10-over	*0.50*		**6.95**	**0.0083**	*0.0077*		*1.09*	**−1.70**	
%change	*1.91*		**7.79**	**2.85**	*2.74*		*4.06*	**−3.11**	
10-over spell, participant 2 (wrist-spin sidespin)									
Mean	28.43	21.12	80.18	0.2821	0.2238	0.1960	24.60	64.86	16.13
RMSE	1.34	3.08	14.16	0.0213	0.0173	0.0291	2.85	4.17	1.12
RMSE% (= CV_RMSD_)	4.72	14.73	17.72	7.56	7.70	15.04	11.61	6.40	6.91
R^2^	0.0661	0.0495	0.0178	0.0702	0.0828	0.0531	0.043	0.0415	0.2058
p-value (alpha = 0.1)	*0.1008*	0.1576	0.4004	**0.0895**	**0.0647**	0.1415	0.1878	0.1943	**0.0025**
Trend of regression	*Increase*	nil	nil	**Increase**	**Decrease**	nil	nil	nil	**Decrease**
Effect on performance	*Gain*	nil	nil	**Gain**	**Loss**	nil	nil	nil	**Gain**
Δ 10-over	*1.75*			**0.0286**	**−0.0254**				**−2.80**
%change	*6.14*			**10.14**	**−11.36**				**−17.34**
10-over spell, participant 3 (finger-spin topsidespin)									
Mean	21.23	42.35	64.55	0.2410	0.2205	0.1200	17.75	50.92	21.22
RMSE	1.54	5.03	7.04	0.0204	0.0221	0.0115	2.89	6.04	0.83
RMSE% (= CV_RMSD_)	7.22	11.89	10.91	8.47	10.01	9.54	16.18	11.91	3.91
R^2^	0.0534	0.013	0.0006	0.0459	0.0732	0.0031	0.0508	0.4316	0.0764
p-value (alpha = 0.1)	**0.0755**	0.3879	0.8500	*0.1003*	**0.0366**	0.6760	**0.0837**	**0.000**	**0.0326**
Trend of regression	**Decrease**	nil	nil	*Decrease*	**Decrease**	nil	**Decrease**	**Increase**	**Decrease**
Effect on performance	**Loss**	nil	nil	*Loss*	**Loss**	nil	**Loss**	**Gain**	**Gain**
Δ 10-over	**−1.26**			*−0.0154*	**−0.0213**		**−2.30**	**18.08**	**−0.82**
%change	**−5.91**			*−6.39*	**−9.68**		**−12.96**	**35.52**	**−3.85**

**Table 4:** Statistics of performance parameters before and after training intervention; avg = average; std = standard deviation).

	Spin Rate *ω* (rps)	Maximum Precession *p* (rad/s)	Maximum Normalised Precession *p_n_* (deg)	Maximum Resultant Torque *T_R_* (Nm)	Maximum Spin Torque *T_s_* (Nm)	Maximum Precession Torque *T_p_* (Nm)	Maximum Angular Acceleration *α* (rad/s^2^)	Maxi-mum Power *P* (W)	Effici-ency *η* (%)	Ratio α_max_ / ω_max_ (s^–1^)	Minimum *T_s_* (Nm) Before Peak Datum	Average *T_p_* (Nm) of a 0.123 s Window (cf. Figure 10)
Profiling, before the training intervention												
Avg	28.31	40.14	94.69	0.3309	0.3172	0.1405	4079	32.38	39.09	22.93	−0.0055	0.0428
Std	0.8	2.66	8.82	0.0112	0.0091	0.0183	117.2	1.81	4.24	0.23	0.0104	0.0065
Min	27.18	35.01	86.91	0.3156	0.3052	0.1143	3925	30.23	34.13	22.64	−0.0223	0.0346
Max	29.23	42.51	108.51	0.3463	0.3307	0.1601	4253	34.94	45.75	23.2	0.0033	0.0492
After the training intervention												
Avg	24.97	38.3	78.26	0.2781	0.2674	0.1234	3439	24.66	58.83	21.91	0.0092	0.027
Std	0.78	2.19	6.07	0.0107	0.0111	0.0081	143.3	1.71	1.75	0.33	0.0042	0.0032
Min	23.8	34.54	71.53	0.2603	0.2488	0.1106	3200	21.96	57.02	21.4	0.0006	0.022
Max	25.84	40.95	89.45	0.2911	0.2816	0.1333	3622	26.41	61.12	22.47	0.0134	0.0307
After several days of self-training												
Avg	27.59	38.33	90.39	0.3197	0.3058	0.1339	3932	30.76	55.2	22.68	−0.0003	0.0296
Std	0.73	1.08	4.47	0.0139	0.014	0.0027	179.9	1.82	2.07	0.45	0.0042	0.0015
Min	26.2	36.58	85.12	0.2953	0.2806	0.1298	3608	27.4	52.12	21.92	−0.0055	0.0269
Max	28.38	39.49	96.3	0.3386	0.324	0.1379	4167	33.04	58.79	23.36	0.005	0.0309

## Data Availability

The data presented in this study are available on request from the corresponding author to any qualified researcher, if they have obtained Ethics Approval for secondary use of existing data through a Consent Waiver.
